# Bidirectional causality between idiopathic sudden sensorineural hearing loss and depression: a Mendelian randomization study

**DOI:** 10.1038/s41598-024-65966-6

**Published:** 2024-06-28

**Authors:** Chuanyu Wu, Ya Yu, Tongtong Zhao, Hui Xie

**Affiliations:** 1 Department of Otorhinolaryngology, Hospital of Chengdu University of Traditional Chinese Medicine, Chengdu, 610072 China; 2 College of Clinical Medicine, Chengdu University of Traditional Chinese Medicine, Chengdu, 610075 China

**Keywords:** Mendelian randomization, Idiopathic sudden sensorineural hearing loss, Depression, Genome-wide association studies, Single nucleotide polymorphism, Computational biology and bioinformatics, Genetics

## Abstract

Idiopathic Sudden Sensorineural Hearing Loss (ISSHL) is a sudden onset, unexplained sensorineural hearing loss. Depression is a common mental disorder and a leading cause of disability. Here, We used a two-sample Mendelian randomization approach using pooled statistics from genome-wide association studies of ISSHL (1491 cases, 196,592 controls) and depression (23,424 cases, 192,220 controls) in European populations. This study investigated the bidirectional relationship between single nucleotide polymorphisms associated with depression and ISSHL using inverse variance weighting.Additional sensitivity analyses, such as Mendelian randomization-Egger (MR-Egger), weighted median estimates, and leave-one-out analysis, were performed to assess the reliability of the findings. Significant causal association between genetic susceptibility to ISSHL and depression in a random-effects IVW approach (OR = 1.037, 95% CI = 1.004–1.072, *P* = 0.030). In contrast, genetic depression was not risk factors for ISSHL (OR = 1.134, 95% CI = 0.871–1.475, *P* = 0.350). After validation by different MR methods and the sensitivity analysis, all of the above results are consistent. The evidence we have gathered suggests a causal relationship between ISSHL and depression. The presence of the former induces or further exacerbates the latter, whereas a similar situation does not exist when the latter is an influencing factor.

## Introduction

Idiopathic sudden sensorineural hearing loss (ISSHL) refers to a rapid and unexplained decrease in sensorineural hearing by at least 30 decibels across three or more frequencies. It may be accompanied by tinnitus, a feeling of dullness in the ears, or symptoms of vertigo, and typically occurs within a three-day timeframe. Despite the high rate of spontaneous recovery from ISSHL, some patients will still have residual symptoms of varying severity or no recovery at all^1,2^. ISSHL occurs in approximately 5 to 20 per 100,000 people worldwide, and the World Health Organization (WHO) has estimated that by 2050, the number of people suffering from varying degrees of hearing loss will reach 2.5 billion globally^3^. At the same time, hearing loss is still the most common cause of disability worldwide,not only seriously affecting the quality of life of patients, but also being the third leading cause of productivity loss over the years^4^.

Depression is a common and treatable mental disorder that is characterised by a pervasive low mood, anxiety, cognitive impairment and somatic symptoms^5^. It is a chronic disease with a worldwide prevalence of depression of around 10%–20%^6^. The prevalence of depression was 18% in men and 26% in women^7^. And almost one in six individuals are expected to develop depression during their lifetime, which is a leading cause of disability^8^.

During an eleven-year observational study^9^, it was discovered that ISSHL had a higher occurrence rate of affective disorders, specifically depression and anxiety. In addition, in a population-based cohort study, depression was identified as a risk factor for ISSHL, which is the most common stress-related disorder^10^. However, it remains uncertain whether there is a correlation between the two.

Mendelian randomization (MR) is a developing approach in genetic epidemiology that utilizes genetic variation as instrumental variables (IVs) to comprehensively understand the causal effect of exposure on outcome^11^. Because genes are randomly assigned during meiosis, this approach eliminates other potential confounders and reverse causality interference, resulting to more meaningful causal conclusions than traditional observational studies^12^.

Consequently, our research employed the methodological framework of Mendelian randomization to investigate the bidirectional causality between the aforementioned factors, adopting an epidemiological standpoint.

## Method

### Study design

**Figure 1:**
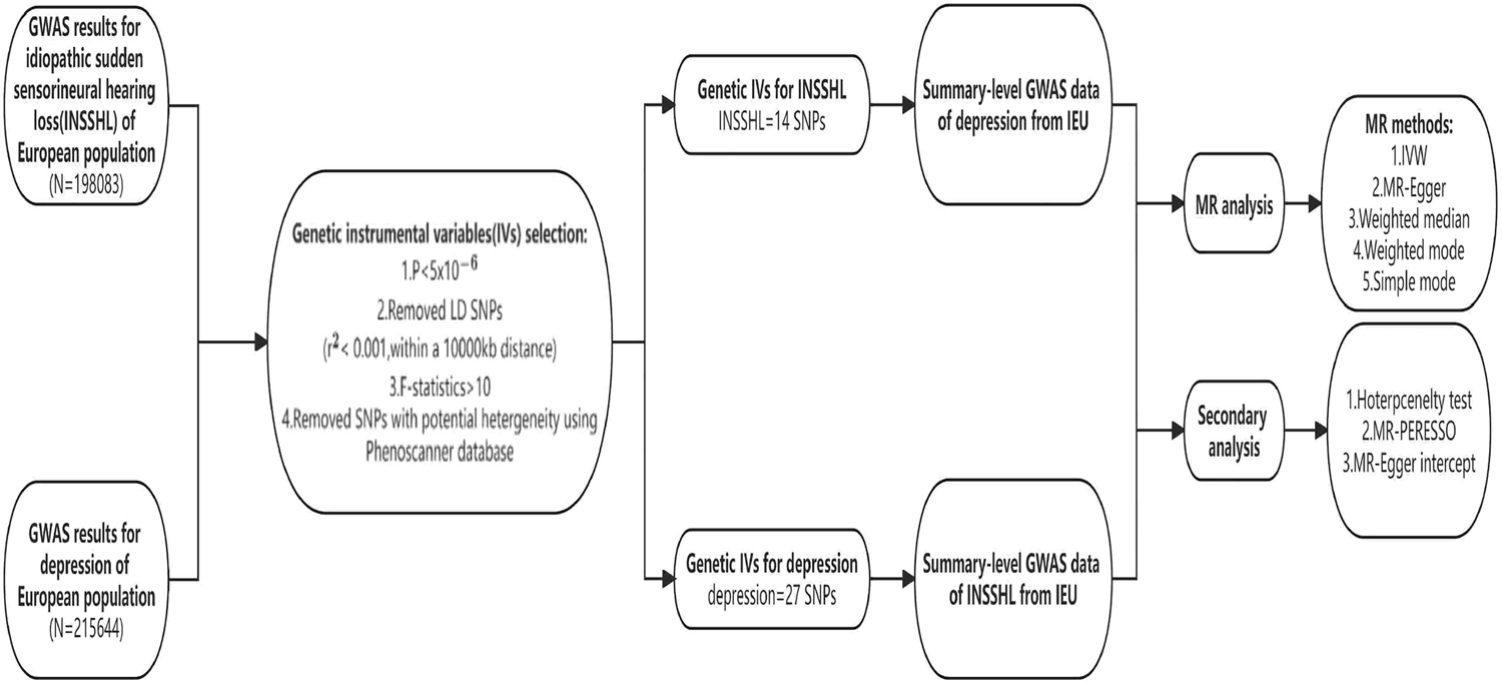
Schematic representation of the Mendelian randomization study design process for depression and ISSHL.

We examined the reciprocal cause-and-effect relationship between ISSHL and depression through a two-sample MR analysis. A persuasive MR design should rest on three fundamental assumptions: (1) the existence of a direct and robust connection between genetic variation and exposure; (2) the notion that genetic variation remains unaffected by potential confounding factors; (3) genetic variation solely influences outcomes through exposure, disregarding other pathways^13^. Figure. 1 presents an overview of this MR methodology. All data utilized in this research were openly accessible and solely limited to the European populace, ensure appropriate ethical approval and informed patient consent for all participants involved in the above studies.

### GWAS data sources

We used a two-sample Mendelian randomization analysis to estimate the causal relationship between depression and ISSHL. This analysis was based on the genome-wide association between the exposure and the outcome. We then transformed this causal relationship to determine the extent to which the latter effect influenced the former. The genetic data for this study were obtained from the IEU database (https://gwas.mrcieu.ac.uk/) for a European population consisting of 1,491 cases with ISSHL and 196,592 controls, another population of 23,424 cases with depression and 192,220 controls. Initially, we chose SNPs (*p* < 5E-8) as the instrumental variable to achieve genome-wide significance. However, the limited number of SNPs obtained prevented the study from proceeding further and, therefore, we loosened the association threshold with *p* < 5E-6 when selecting SNPs. We selected the independent variants linked to depression-related traits by utilizing the PLINK clump command, employing thresholds below *P* < 5E-6, and analyzing data from European populations. Additionally, for linkage disequilibrium, we used a threshold of r2 < 0.001 at a distance of 10,000 kb. Finally, we calculated the F statistic for each SNP: F = $\frac{{{\text{beta}}^{{2}} }}{{{\text{se}}^{{2}} }}$^14^. In this process, we selected SNPs with F > 10 to ensure that each SNP was strongly correlated with exposure.

A two-sample Mendelian randomization analysis was conducted to estimate the causal relationship between ISSHL and depression, by examining genome-wide associations between the exposure and outcome.

We ultimately examined 14 ISSHL loci. Initially, ISSHL was regarded as the exposure, depression was identified as the outcome, and SNPs were treated as instrumental variables. The same approach was then used to select independent variants associated with the trait of depression, resulting in 27 loci that were considered as exposure, ISSHL as outcome, and SNPs as instrumental variables.

### Estimation of Mendelian randomization

We used the inverse variance weighed (IVW) to weight the estimated effects of each SNP on sudden idiopathic hearing loss and depression^13^. The goal of this weighted linear regression model is to combine and minimize the total variances. Meta-analytic models, including fixed-effects and random-effects, can be utilized to estimate the impact of genetically related ISSHL on the likelihood of developing depression. Again, we performed the same operation after exchanging the identities of ISSHL and depression.

### Genetic pleiotropy evaluation

Mendelian randomization-Egger (MR-Egger) regression method is frequently employed in meta-analyses to assess publication bias and is robust against multidirectionality^15^, assuming that the instrument strength is unrelated to direct effects. We used this approach to evaluate the hypothesis and detect multidirectional effects on the target gene. The MR-Egger regression slope was also employed in estimating the causal impact of multidirectionality. This is sufficient if the selected SNPs account for the proportional difference and the estimates of exposure effects by SNPs must be independent of their direct effect on the outcome. The MR-Egger has a tendency to yield dependable causal estimates, even in scenarios where the chosen SNPs are not strong. In order to estimate causal effects more reliably, weighted median^16^, weighted mode^17^, and simple mode method was used to complement MR-Egger. In addition to the above, we also performed MR-Egger intercept tests to test for the presence of an unbalanced lateral pleiotropy. If pleiotropy exists, then the analysis results will show a p-value of the intercept of less than 0.05^15^.

The MR-PRESSO method has the capability to identify and eliminate outliers, leading to more reliable and unbiased estimates^18^. To ensure meaningful predictions, we employed Cochrane Q-values to evaluate heterogeneity, and it did not exist if the analytical outcome P-value was greater than 0.05^15^.

All statistical analyses were performed using R 4.3.0 and Mendelianrandomization version 0.5.7 for Windows.

### Sensitivity analyses

To ensure that the Mendelian randomisation study was not confounded by confounding factors, we screened the SNPs using Phenoscanner and found that none of the SNPs were associated with confounding factors. We further conducted leave-one-out analyses to explore potential SNP-driven associations^19^.

### Logistic regression

We analyzed the hearing and depression survey data from the NHANES database to enhance the robustness of our results. Due to the insufficient sample size for 2017–2018, analysis was not feasible, leading us to utilize the population survey data from 2015–2016. Lacking independent data specifically related to ISSHL, we used hearing loss as a surrogate. For the two variables under study, the selection criteria were as follows: concerning audiometric data, we considered all frequencies with values below 25 decibels as indicating no hearing loss; any frequency with a value above 25 decibels was considered indicative of hearing loss. Regarding the depression survey data, the NHANES database employed the PHQ-9 questionnaire. Therefore, we determined that a total score of 0–9 indicated no depression, while a score of 10–27 indicated the presence of depression when each item was meaningful.

After incorporating variables such as age, gender, race, and marital status, we obtained 3951 valid data points, as detailed in Table 1. Subsequently, we treated depression as the dependent variable and the others as independent variables, and conducted logistic regression analysis.

**Table 1:** Results of the screening of relevant data for 2015–2016.

	Depression		*P*
	Absent	Present	
*N* = 3951	3625	326	
Age	44.00 (32.00–57.00)	48.00 (35.00–59.00)	0.033
Gender			0.001
Male	1791 (49.41%)	131 (40.18%)	
Female	1834 (50.59%)	195 (59.82%)	
Race			< 0.001
Mexican American	688 (18.98%)	49 (15.03%)	
Other hispanic	485 (13.38%)	50 (15.34%)	
Non-hispanic white	1089 (30.04%)	124 (38.04%)	
Non-hispanic Black	795 (21.93%)	71 (21.78%)	
Non-hispanic Asian	434 (11.97%)	15 (4.60%)	
Other race—including multi-racial	134 (3.70%)	17 (5.21%)	
Marital status			< 0.001
Married	1911 (52.72%)	109 (33.44%)	
Widowed	104 (2.87%)	10 (3.07%)	
Divorced	352 (9.71%)	50 (15.34%)	
Separated	111 (3.06%)	26 (7.98%)	
Never married	736 (20.30%)	95 (29.14%)	
Living with partner	410 (11.31%)	36 (11.04%)	
Refused	1 (0.03%)	0 (0.00%)	
Hearing loss			0.002
Absent	1542 (42.54%)	110 (33.74%)	
Present	2083 (57.46%)	216 (66.26%)	

## Results

**Figure 2:**
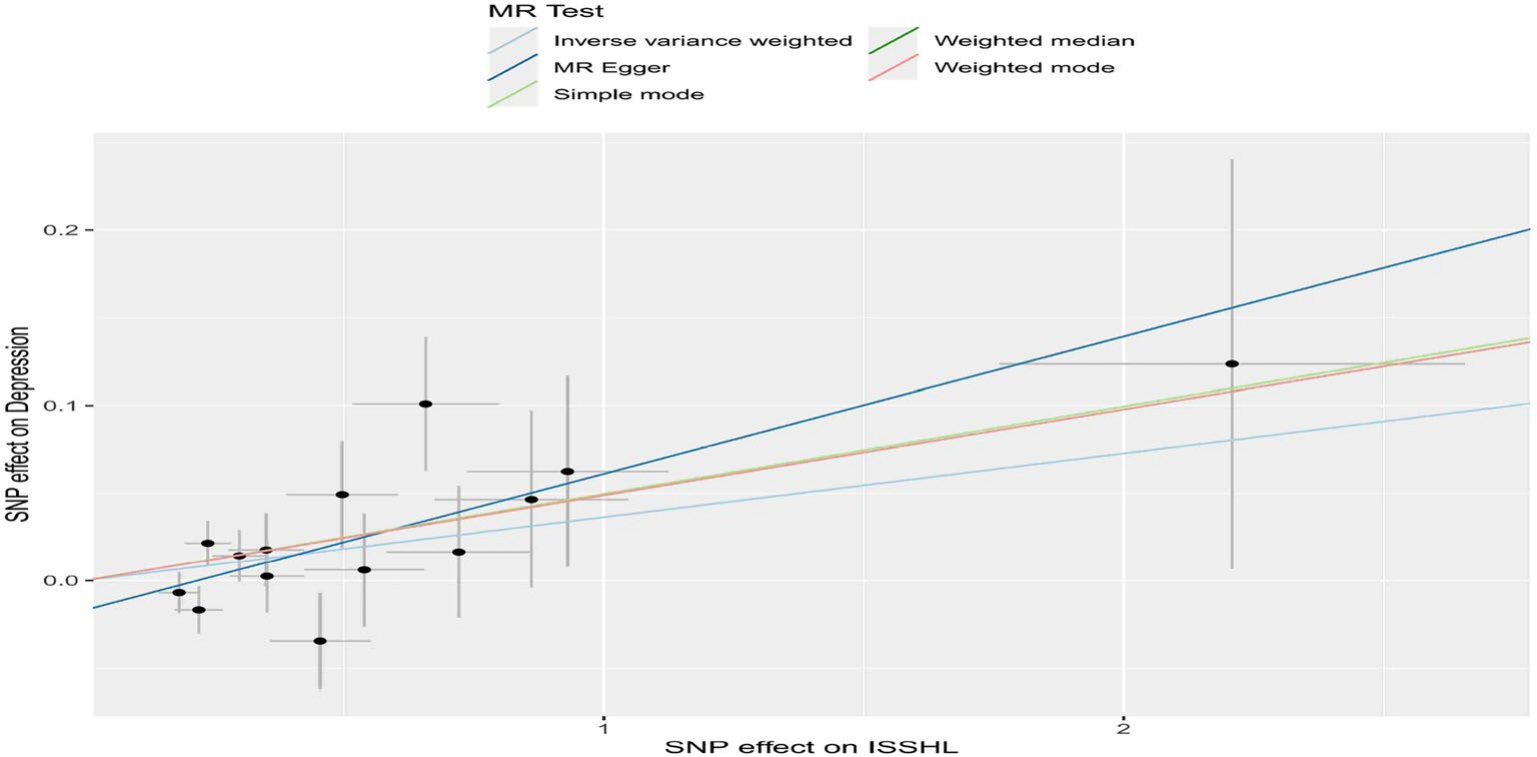
Scatter plot of the potential effects of ISSHL associated SNPs on depression.

**Figure 3:**
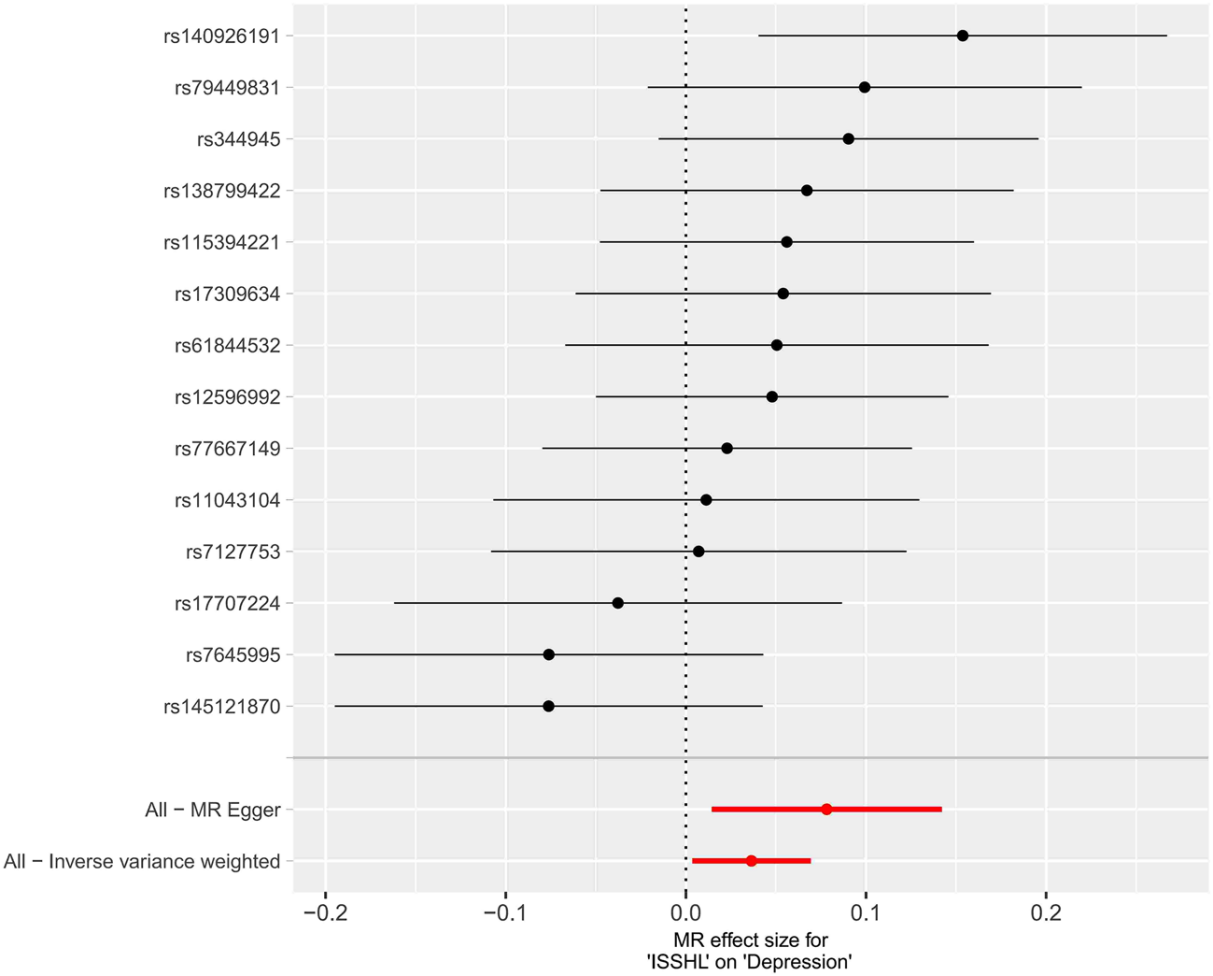
Forest plot of the potential effects of ISSHL associated SNPs on depression.

**Figure 4:**
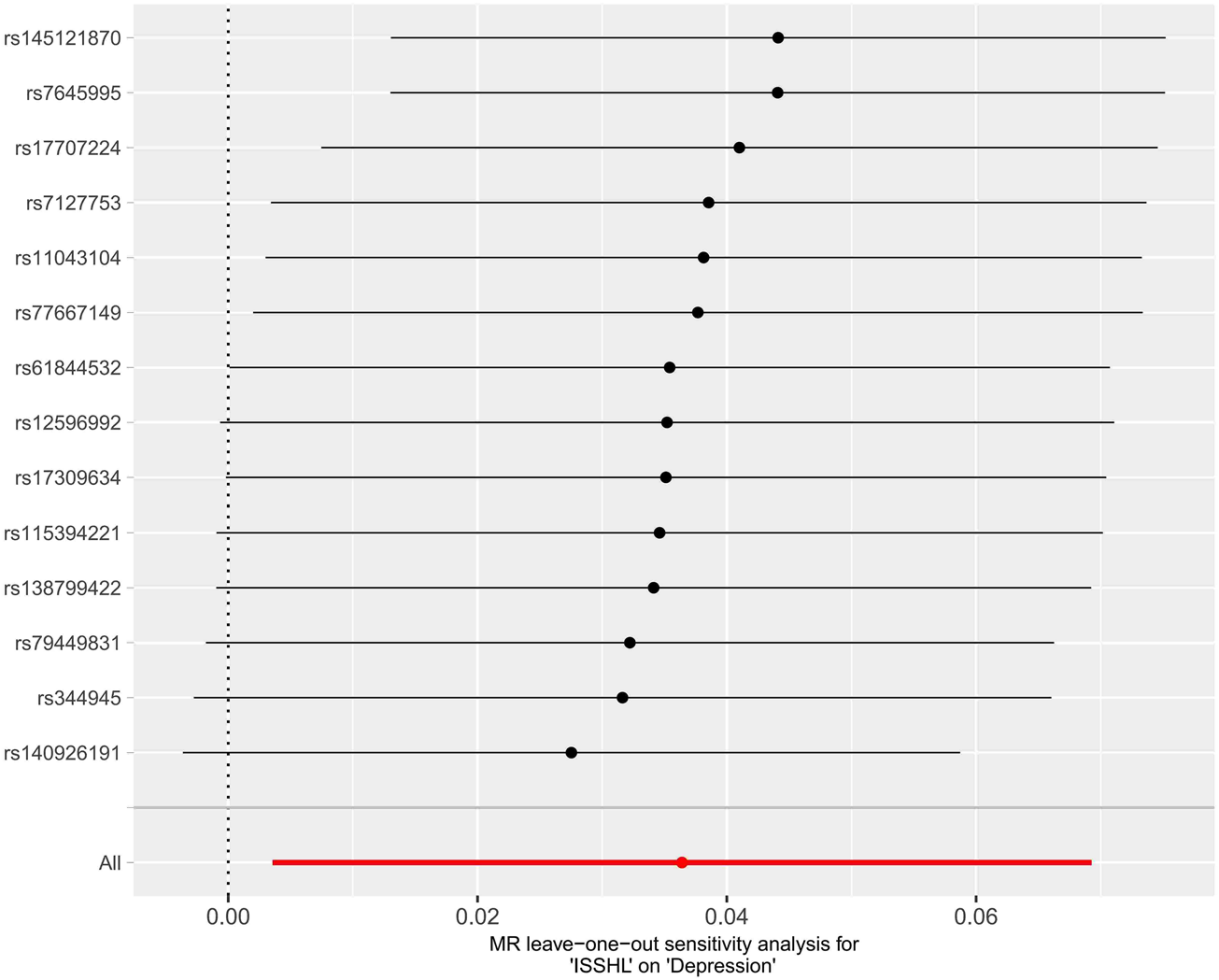
Leave-one-out plots for the MR analyses of ISSHL on depression.

**Figure 5:**
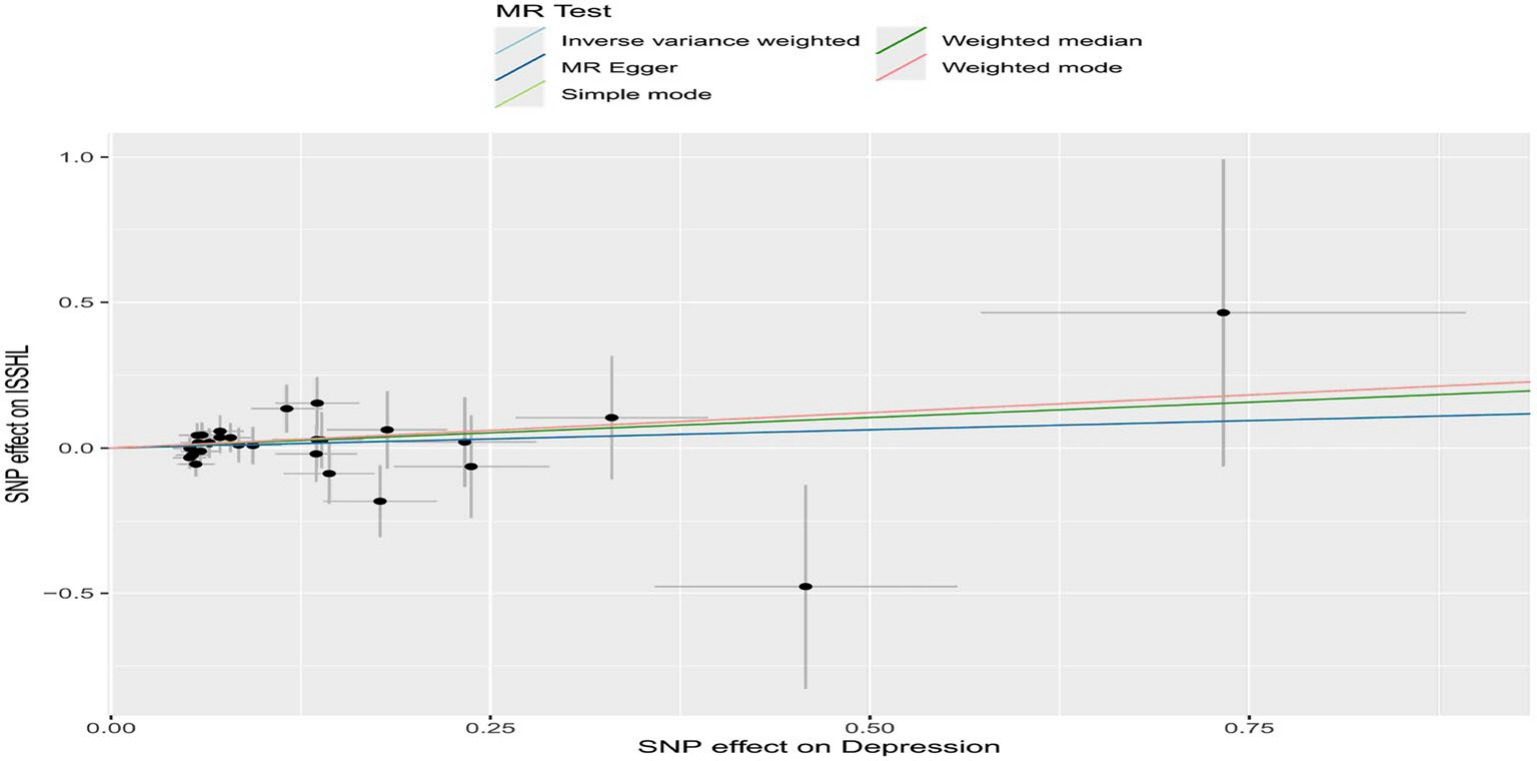
Scatter plot of the potential effects of depression associated SNPs on ISSHL.

**Figure 6:**
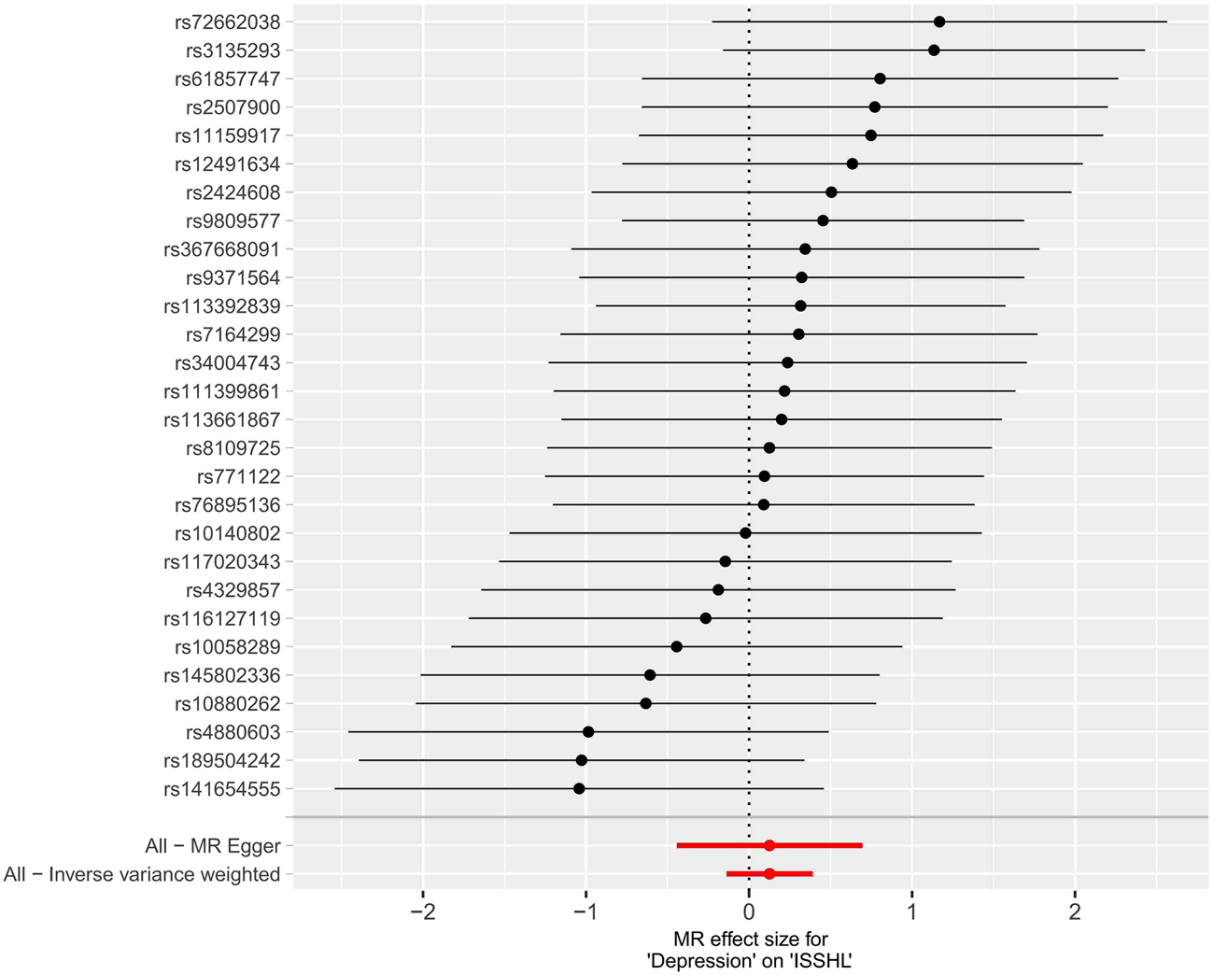
Forest plot of the potential effects of depression associated SNPs on ISSHL.

**Figure 7:**
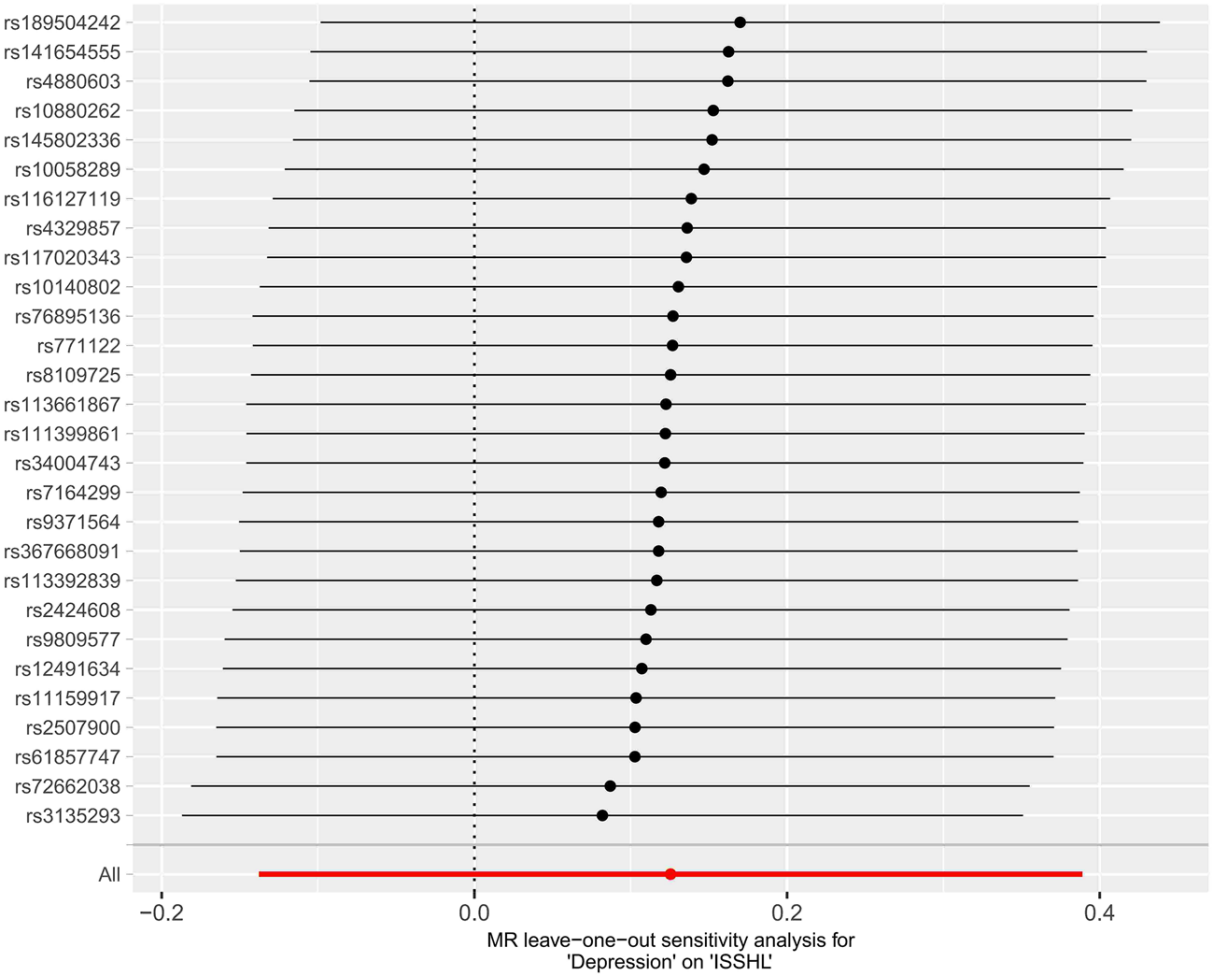
Leave-one-out plots for the MR analyses of depression on ISSHL.

To examine the relationship between the effect of SNPs on exposure (ISSHL) and the effect of SNPs on the outcome (depression), we compared standard IVW analysis and MR-Egger analysis with potential horizontal multidirectional correction. Our findings are presented in Figs. 2, 3, 4 and Table 2. Additionally, we conducted an analysis using the same method after interchanging the causality of the two diseases. The results are depicted in Figs. 5, 6, 7 and Table 3.

**Table 2:** MR analyses showing the associations of genetically ISSHL with the risk of depression.

Method	BETA	SE	*P*	OR	95% CL
Inverse variance weighted	0.036	0.017	0.030	1.037	(1.004,1.072)
MR Egger	0.078	0.033	0.033	1.081	(1.015,1.153)
Weighted median	0.049	0.022	0.023	1.050	(1.007,1.095)
Simple mode	0.050	0.039	0.219	1.051	(0.975,1.133)
Weighted mode	0.049	0.036	0.199	1.050	(0.978,1.127)

**Table 3:** MR analyses showing the associations of genetically depression with the risk of ISSHL.

Method	BETA	SE	*P*	OR	95% CL
Inverse variance weighted	0.125	0.134	0.350	1.134	(0.871, 1.475)
MR Egger	0.125	0.290	0.669	1.134	(0.642, 2.002)
Weighted median	0.210	0.189	0.268	1.233	(0.851, 1.787)
Simple mode	0.243	0.333	0.472	1.275	(0.664, 2.448)
Weighted mode	0.243	0.359	0.504	1.275	(0.631, 2.577)

In summary, 14 ISSHL initiation-related SNPs were found to be significantly associated with depression (IVW estimated odds OR = 1.037, 95% CI 1.004–1.072, *P* = 0.030). However, no statistically significant connections were observed between ISSHL and the 27 depression initiation-related SNPs (IVW estimated odds ratio OR = 1.134, 95% CI 0.871–1.475, *P* = 0.350).

In the two instances where ISSHL and depression were found to be causally linked, the intercept analyses conducted using MR-Egger indicated no statistically significant results. This suggests that genetic pleiotropy did not contribute to any bias in causation. Additionally, the leave-one-out analyses showed that the majority of correlation signals were not influenced by a single genetic marker (Fig. 4 and Fig. 7).

The logistic regression analysis (Table 4) revealed that hearing loss, whether as an univariable (OR = 1.45, 95% CI 1.14–1.85, *p* = 0.002) or as a multivariable (OR = 1.48, 95% CI 1.10–1.99, *p* = 0.010), is a risk factor for depression.

**Table 4:** Logistic regression analysis with depression as the dependent variable.

Dependent: Depression		Absent (*N* = 3625)	Present (*N* = 326)	OR (univariable)	OR (multivariable)
Gender	Male	1791 (49.4%)	131 (40.2%)	Baseline	Baseline
	Female	1834 (50.6%)	195 (59.8%)	1.45 (1.15–1.83, *p* = 0.002)	1.47 (1.15–1.86, *p* = 0.002)
Age	Mean ± SD	44.4 ± 14.3	46.2 ± 14.4	1.01 (1.00–1.02, *p* = 0.033)	1.01 (1.00–1.02, *p* = 0.105)
Race	Mexican American	688 (19%)	49 (15%)	Baseline	Baseline
	Other Hispanic	485 (13.4%)	50 (15.3%)	1.45 (0.96–2.18, *p* = 0.078)	1.28 (0.84–1.94, *p* = 0.249)
	Non-hispanic white	1089 (30%)	124 (38%)	1.60 (1.13–2.26, *p* = 0.007)	1.58 (1.12–2.25, *p* = 0.010)
	Non-hispanic black	795 (21.9%)	71 (21.8%)	1.25 (0.86–1.83, *p* = 0.241)	1.04 (0.71–1.54, *p* = 0.839)
	Non-hispanic Asian	434 (12%)	15 (4.6%)	0.49 (0.27–0.88, *p* = 0.016)	0.56 (0.31–1.02, *p* = 0.057)
	Other race—including multi-racial	134 (3.7%)	17 (5.2%)	1.78 (1.00–3.19, *p* = 0.052)	1.69 (0.93–3.05, *p* = 0.083)
Martial status	Married	1911 (52.7%)	109 (33.4%)	Baseline	Baseline
	Widowed	104 (2.9%)	10 (3.1%)	1.69 (0.86–3.32, *p* = 0.131)	1.21 (0.60–2.40, *p* = 0.596)
	Divorced	352 (9.7%)	50 (15.3%)	2.49 (1.75–3.55, *p* < .001)	2.07 (1.44–2.97, *p* < .001)
	Separated	111 (3.1%)	26 (8%)	4.11 (2.57–6.56, *p* < .001)	3.74 (2.32–6.04, *p* < .001)
	Never married	736 (20.3%)	95 (29.1%)	2.26 (1.70–3.02, *p* < .001)	2.78 (2.02–3.83, *p* < .001)
	Living with partner	410 (11.3%)	36 (11%)	1.54 (1.04–2.28, *p* = 0.031)	1.73 (1.15–2.59, *p* = 0.008)
	Refused	1 (0%)	0 (0%)	–	–
Hearing loss	Absent	1542 (42.5%)	110 (33.7%)	Baseline	Baseline
	Present	2083 (57.5%)	216 (66.3%)	1.45 (1.14–1.85, *p* = 0.002)	1.48 (1.10–1.99, *p* = 0.010)

## Discussion

In our study, a two-sample MR study based on large-scale GWAS data from the current European population showed that a positive correlation between ISSHL and the risk of depression plays a crucial role, but that there is no positive correlation between depression and ISSHL when the two are causally interchanged. After being validated by multiple sensitivity analyses, these results remain reliable and support our conclusions.

Multiple studies have demonstrated a significant correlation between ISSHL and the likelihood of experiencing affective disorders^20–22^. Partial patients with ISSHL remit or are completely cured, but about 60% or more still have hearing problems^1^. Patients with ISSHL and hearing loss often have hearing difficulties with background noise and recognizing sound sources^23^. Untreated severe hearing loss in these individuals can cause them to become socially isolated, which can negatively impact their mental health compared to the general population^24,25^. Consistent with this hypothesis, a previous study found that untreated ISSHL patients were more likely to experience symptoms of major depression, potentially due to increased emotional distress^26^. A longitudinal epidemiological study involving over one million participants revealed a notable rise in the occurrence of affective disorders among patients with ISSHL; more specifically, the study demonstrated a significantly higher likelihood of developing depression and anxiety disorders due to ISSHL, while bipolar disorder did not show any significant association^9^. Additionally, it is worth noting that tinnitus often accompanies hearing loss in patients with ISSHL^27,28^, and previous research has revealed a higher occurrence of anxiety and depression among individuals with tinnitus who also have comorbid psychological disorders^29,30^.

Yet, the link between ISSHL and depression has not been investigated from the standpoint of genetic epidemiology. Therefore, we obtained the same results as previous related studies by two-sample Mendelian randomization, namely that ISSHL is a risk factor for depression, providing further evidence to support for this view. Our logistic regression analysis demonstrates that hearing loss is a risk factor for depression, whether in univariable or multivariable models. ISSHL is a unique form of auditory impairment characterized by symptoms more severe than those of single-frequency hearing loss. Therefore, we believe that if hearing loss is a risk factor for depression, then ISSHL, as a subset of it, is also a risk factor for depression. This finding is consistent with our Mendelian randomization results, further enhancing its reliability.

Stress refers to the non-specific systemic reaction that occurs when the organism is stimulated by various strong factors (environmental factors, intrinsic factors of the organism, psychological and social factors). The stress theory was firstly proposed by Selye, a Canadian scholar, who believed that any kind of disease or harmful stimulus would trigger physiological and biochemical reactions throughout the body^31^. Therefore, we believe that ISSHL, as a sudden-onset disease of unknown etiology, will have a greater impact on the physiology and psychology of the patient, causing the organism to enter a state of stress. When this state persists, the patient's HPA axis becomes hyperfunctional, resulting in a chronic increase in cortisol (a glucocorticoid) levels^32^. Excessive glucocorticoids cause dendritic atrophy, neurogenic inhibition, and increased neurotoxicity in the glucocorticoid receptor-rich forebrain (especially the hippocampus) and limbic system, resulting in mood and cognitive deficits^33^.

On the other hand, the etiology of ISSHL remains unclear, although otology has made great strides in the last few decades. The lack of conclusive evidence has led to speculative best prevention and treatment methods and an ongoing debate. While approximately 100 potential causes have been identified over time, the majority of cases remain idiopathic, making the prognosis uncertain for each individual^34^.

In recent studies, multiple scholars have documented a relationship between mental health issues and sudden hearing loss^20,26,35^. Some scholars have noted that patients with ISSHL display depressive symptoms, however, they did not distinguish between cause and result. Some individuals discovered that patients recovering from ISSHL had an even higher prevalence of depressive symptoms^26^. Patients with ISSHL often have low levels of natural killer cell activity, which is frequently linked to increased systemic stress^35^. The reciprocal connection between ISSHL and depression implies the presence of shared physiological mechanisms.

According to previous reports, cerebral ischemia has been proposed as a possible cause of ISSHL and has been shown to affect prognosis^36,37^. The cochlea, being an end organ, receives its blood supply from a limited number of branches of the internal auditory artery and does not have any alternative vascular collateral circulation^38^. It has been acknowledged that mental stress is an autonomous risk factor for illnesses related to vascular dysfunction. Depression, the most prevalent stress-related disorder, raises the chance of developing cardiovascular disease in patients by 1.5–2 times^39^. At the same time, the exact ways in which psychosocial stress raises the possibility of vascular insufficiency remain uncertain and could be intricate. Evidence of coronary atherosclerosis and endothelial dysfunction has been found in response to psychosocial stress^40–42^. Furthermore, anxiety and stress can affect the degradation of nitric oxide in the body, resulting in either increased or decreased levels. This modulation of nitric oxide levels plays a vital role in regulating the sensitivity of endothelial cells to platelet and monocyte adhesion by altering the concentrations of cortisol, epinephrine, and norepinephrine in the bloodstream^43^. Hence, stress can result in instability of blood vessel dilation, constriction of blood vessels, and an increased risk of blood clot formation, which can serve as prognostic or even causative factors in ISSHL. However, it was shown through our study that when depression was used as a risk factor, it did not increase the risk of ISSHL or further exacerbate the symptoms of ISSHL, which is contradictory to previous studies.

This study is noteworthy as it is the first genetic-based two-sample MR study to explore the potential causal link between depression and ISSHL. The study's key strength is that we utilized an extensive data set of GWAS data from a European population to examine the reciprocal association between these two disorders. This approach minimized bias resulting from population stratification and ensured our statistical power was strong and persuasive. The two-sample MR design also provides causal evidence that ISSHL is a risk factor for depression, whereas the latter is not significantly associated with the former, largely eliminating the effects of traditional study limitations.

However, there are some limitations to this study. First, the participants in the GWAS who took part in the study were limited to Europe, the data were large, and it remains highly questionable whether the same results can be extrapolated to other ethnic groups. Second, the lack of stratified data such as age and gender in the available summary statistics prevented us from conducting a comprehensive and more detailed analysis. If more detailed and publicly available stratified data become available in the future, this would allow further elaboration of new MR analyses.

## Conclusion

To summarize, this MR study presents initial genetic evidence supporting a causal connection between ISSHL and a heightened risk of depression. However, no evidence was found for a causal relationship between depression and ISSHL. These findings offer further proof that ISSHL heightens vulnerability to depression. ISSHL causes a stress response that may contribute significantly to depressive episodes. There are several prognostic factors associated with the likelihood of hearing recovery in patients with ISSHL in the field of ear science. Since maximizing the chance of hearing recovery is the main objective of treatment, stress management could be included as an essential component of the treatment plan for ISSHL. While depression itself does not elevate the risk of ISSHL, studies indicate that the use of antidepressant drugs could potentially contribute to the development of ISSHL^44^.Therefore, it is important to approach the treatment of both ISSHL and depression with caution, in order to minimize any potential direct or indirect interaction between the two conditions.

## Data Availability

The original contributions presented in the study are included in the article, and the corresponding authors can be contacted directly for further inquiries.
